# The Story of the Insane from Year to Year

**Published:** 1890-10-04

**Authors:** 


### THE STORY OF THE INSANE FROM YEAR TO YEAR.

[Annual Reports, Pamphlets, &c., should be addressed to THE EDITOR The Lodge, Porchester Square, London, W.]

### BERKS COUNTY ASYLUM.

THIS asylum was opened towards the close of the year 1870, and at the end of 1871 it contained 248 patients. At the end of 1889 the numbers had risen to 502, showing that the insane population of the asylum had been more than doubled during the year. There were 97 admissions, 49 recoveries, and 28 deaths during the year. The percentage of recoveries is 57·6, and the death-rate is only 5·5. Like other medical superintendents, Mr. Douty insists on the necessity of early treatment, and he remarks that "no noticeable increase could accrue to the recovery rates of county asylums as they at present stand by the erection of elaborate and expensive hospitals, or by any other conceivable means, whilst so many cases are kept at home until all possibility of recovery has passed." This is very true, and we may add that the hospital idea has had a fair hearing: has emerged badly from its trial, and is probably bowled over for ever. There was an outbreak of typhoid fever apparently due to impurity of the water. Mr. Douty has a slap at the new Lunacy Act, and beyond doubt the new Lunacy Act deserves the slap. An Amendment Act is one of the wants of the immediate future, or rather of the present.

### NOTTINGHAM COUNTY ASYLUM.

This asylum contains only 300 patients, and is thus the second smallest of our county asylums. It is also one of the oldest, and according to the Commissioners in Lunacy, it ought to be provided with a chapel, a recreation hall, a dining hall, additional water closets, a visiting room for patients' friends, attendants' mess rooms, means of communication between the blocks and the medical officers' quarters, modern baths, removal of the concrete floors, and new workshops. These would now-a-days be called "a large order." Although the Commissioners recommend these things, and indeed urge the erection of an entirely new asylum, they say that "the wards and dormitories now occupied by patients were in good order, and the patients remarkably quiet." This shows that Dr. Aplin makes the most of the poor old asylum. The visitors say that "the management of the asylum, the conduct of the officers and servants, and the care of the patients are such as merit the approval of the committee."

### EARLSWOOD ASYLUM FOR IDIOTS.

This institution contained at the close of the year 599 inmates. During 1889 64 fresh cases were admitted, 40 were discharged, and 32 died. Dr. Jones thinks the causes of congenital imbecility remain among the unexplained mysteries of nature, and we entirely agree with him; at the same time we hope he will not fall down powerless before the mystery, for few living men are better able to make investigations on the subject. "It has frequently been stated," says Dr. Jones, "that the oldest child is more often imbecile than others in a family, and considering the influence of maternal impressions and the exalted emotional life of a young mother under new circumstances such might almost have been expected. It would also be expected that boys should be affected in a larger relative proportion than girls, but neither is the case." The asylum is conducted in great part on charitable lines, but paying cases are admitted. The Commissioners in Lunacy remark that "credit is due to the staff for the cleanly and happy appearance of the patients." The committee say that the "helpless class committed to their charge has been carefully and kindly tended during the past year under the able management of the Medical Superintendent, Dr. Robert Jones, and his assistant, Mr. E. F. Cooper.

